# T117. INVESTIGATION OF FORMAL THOUGHT DISORDER AND RESPONSE TO TREATMENT IN SCHIZOPHRENIA

**DOI:** 10.1093/schbul/sby016.393

**Published:** 2018-04-01

**Authors:** Fernando Rocha Loures Malinowski, Bruno Bertolucci, Cristiano Noto, Deyvis Rocha, Cinthia Higuchi, Rodrigo Bressan, Ary Gadelha

**Affiliations:** Federal University of Sao Paulo (UNIFESP)

## Abstract

**Background:**

Formal thought disorder (FTD) is a multidimensional dysfunction characterized by inability to maintain a coherent speech in spoken or written language, poor social cognition and disorganized thought itself.

Presence of formal thought disorder has been associated with poor prognosis in schizophrenia, but the association with treatment response is yet to be determinate. Formal thought disorder has a close relation to disorganized symptoms in schizophrenia, which were independently associated with treatment resistance and poor response to standard antipsychotics. Formal thought disorder investigation could provide a clinical construct better delimited to assess disorganized symptoms in schizophrenia.

We investigated the association between FTD, remission and treatment resistance in patients with schizophrenia.

**Methods:**

This study reunite a sample of 213 patients, between 14 and 69 years, who met DSM-IV criteria for schizophrenia.

The analyses were conducted in two samples conducted independently. In both samples, Diagnostic evaluation was performed with the Structured Clinical Interview for DSM-IV Axis I Disorders (SCID-I), response to treatment was primarily assessed through PANSS, functional impairment was assessed by GAF and disease severity, by CGI. The first sample was a follow-up study that enrolled inpatients. Participants were rated at baseline and after four weeks of antipsychotic treatment. If the participant did not reduce a minimum of 40% of baseline PANSS, the antipsychotic was switched. If the participant did not reduce a minimum of 40% in total PANSS in the following antipsychotic trial, the participant was considered as treatment resistant schizophrenia (TRS) and clozapine, introduced. The second sample was enrolled in an outpatient clinic specialized in schizophrenia.

Illness remission was defined as a severity of mild (score of 3 on a scale of 1 to 7) or less for P1, P2, P3, G9, G5, N1, N4 and N6 PANSS`s items.

To stablish FTD severity, PANSS items related to high scores at the Thought and Language Index (TLI) were considered: P2, P6, N1, N2, N5, N6, G7 and G9.

**Results:**

Most subjects were male (56.8%) and the mean age was 34.42 (±12.33 SD).

The FTD failed to associate with remission (t = 4.007, p = 0,491) or treatment resistance (t = -3.768, p = 0.988) in both samples.

FTD had a negative correlation with GAF (r = -0.473, p<0.01) and a positive correlation with CGI (r = 0.530, p<0.01).

**Discussion:**

FTD had a stronger association with global functioning and severity measures, rather than treatment symptomatic outcomes. In future studies, we will investigate whether FTD show distinctive clinical features commonly related to disorganized syndrome, i.e. earlier age of onset.

